# Everybody's Page

**Published:** 1888-09-15

**Authors:** 


### Everybody's Page.

The average height of Frenchmen is 5 ft. 4 in.; their average weight, 134 lb.

Freckles have some relation to the general state of health, and are not solely due to the action of the sun. Many people have plenty of them on parts of the body that are not exposed to its rays,

Strong emotions have a powerful influence on digestion. Anxiety and depression need not be extreme, but merely pretty constant for a while, to produce dyspepsia, especially in people of a sanguine temperament.

Clothes sent from one place to another may convey disease a great distance. It was in this way that Eyam, in Derbyshire, was nearly depopulated by the plague, the infection of which was conveyed in some cloth sent from London, where the plague was raging, to a tailor in that isolated and picturesque village.

Dr. Greenhow, in his inquiry into the causes of lung disease, mentions amongst those who suffer most from this cause the grinders of Sheffield and Birmingham, the brassworkers of these towns and Wolverhampton, the tinmen and enamellers of hardware and button-makers of Birmingham, and the card-room and cotton and weaving operatives of Nottingham and other places.

During the whole period of childhood and growth it is scarcely possible to have food better suited to the wants of the body than good well-cooked oatmeal porridge with milk. But it is most important that oatmeal and all farinaceous food should be thoroughly well cooked. It should be boiled for a good half-hour or more. And as children like sweets, they seldom refuse some good treacle with it.

John Howard, the philanthropist, says that in his day "the malignity of the air in gaols" was such that in his first journey his clothes were so offensive that he could not bear the windows of a post-chaise up, and was often obliged to travel on horseback, and the leaves of his note-book were so offensive that he could not use it until it had been open for an hour or two before the fire.

Drunkards are always dyspeptic ; and even those whose habits fall far short of what would be called drunkenness suffer sooner or late. The dyspepsia produced by stimulants is usually of the acute sort—that is, it is produced by irritation leading to inflammation of the lining membrane of the stomach, and, according to the amount of mischief done, accompanied by more or less severe symptoms.

When the family of Louis Philippe, ex-King of France, resided in this country at Claremont, they suffered from lead poisoning, and it was found that the amount of lead did not exceed one grain in a gallon; and probably one-tenth, or even less, per gallon is sufficient to be dangerous. Lead colic was formerly very prevalent in Devonshire, and it was found to be due to the presence of lead in the cider, which is the common drink of that country. This arose from its being used in the construction of the cider troughs.

Carbonic acid gas is the deadly vapour that is given off when charcoal is burnt in a close room. It is the choke-damp of the miner, and the gas that collects in old pits and wells, and that pours out into the Grotto del Cane, near Naples. Any human being or animal entering into these reservoirs of the gas is as surely suffocated as if he were immersed in water. It is in truth a poisonous compound, and only very small proportions of it in the air can be breathed without injury to health

There are very few bed-rooms that will stand the test of leaving them for the outdoor atmosphere, and then on returning discovering no closeness or foulness of air. And yet this, especially for the young, is the limit of safety from disease, and especially from lung disease and scrofula.

The stomach is the kitchen of the body. It has to perform the important work of keeping up the health and spirits of the various organs, by properly cooking and preparing their food. This process of cooking, as done in the stomach and its appendages, is called digestion.

It has been calculated that "the lungs of a full-grown man contain 600,000,000 minute blood-vessels, and if the membranes of which they are composed were spread out flat they would cover 21,000 square inches of surface—a space thirty times larger than the external skin." Lavishly spread over the surfaces of this membrane are fine hair-like blood-vessels, twisted and coiled about so as to offer as much surface as possible, and at the same time permit the distension of the air-cells without stretching or breaking any one of them.

In 1854, at some of the stations of the Austrian army in Hungary, the plan was commenced of treating a portion of the patients in tents instead of in the permanent hospitals. The results were very satisfactory. "The most severe maladies ran their course" much more mildly in the free air, and the patients recovered more quickly and more perfectly than in the confined spaces of hospitals. Typhoid fever, small-pox, wounds, and other inflammations did much better in the tents; and in no single case could it be made out that gangrene originated in a tent.

Fifty-five years ago, a Canadian, named St. Martin, was accidently shot through the stomach. A part of the front wall remained open, but the man recovered and retained quite good health. By pushing aside a sort of valve that formed to close the opening, the inside of the stomach could be seen, and the process of digestion observed. Observations and experiments were made upon this man for several years, and a great deal of information was obtained concerning the process of digestion in the stomach, and the circumstances that aid or hinder it.

Bromide of potassium taken in large doses before sailing, and in small doses after, is said to be one of the best remedies for the prevention of sea-sickness; but it is not a drug for anyone to prescribe for himself. Another plan which is thought to be infallible is to rub the head immediately at the back of the ears until the skin is slightly excoriated, as this excites a certain nerve on which it is thought that sea sickness is dependent. Persons who advance this theory for the cure of sea-sickness strengthen their claims to its efficacy by the fact that deaf mutes in whom the nerve thus agitated does not exist, never suffer from this distressing malady.

The influence, both of occupation and foul air, may be seen in the contrast between the male and female rates of mortality from consumption in different districts. In some parts of England the men are the chief workers at indoor employments—as in Sheffield and Birmingham; there you find the male rate the highest; in others, as at Nottingham, Huddersfield, and Macclesfield, the women are mostly employed, and consequently they die most numerously of consumption; and in places like Liverpool, and Manchester, and Stockport, where there is little difference in the employment of men and women, there is also little difference in the rates of mortality from consumption; both are high.

